# Genetic association of serum calcium, phosphate, vitamin D, parathyroid hormone, and FGF23 with the risk of aortic stenosis

**DOI:** 10.1038/s44325-024-00013-x

**Published:** 2024-08-21

**Authors:** Qinghao Zhao, Wenchang Nie, Jiaming Dong, Bowen Zhang, Gongzheng Tang, Shenda Hong, Jian Liu

**Affiliations:** 1 Department of Cardiology, Peking University People’s Hospital, Beijing, China; 2 School of Medicine, Shaoxing University, Shaoxing, Zhejiang China; 3 Department of Radiology, Beijing Chaoyang Hospital, Capital Medical University, Beijing, China; 4 National Institute of Health Data Science, Peking University, Beijing, China; 5 Institute of Medical Technology, Health Science Center of Peking University, Beijing, China

**Keywords:** Valvular disease, Cardiovascular genetics

## Abstract

Disorders of mineral metabolism, including elevated levels of serum calcium, phosphate, 25-hydroxyvitamin D (25OH-VitD), parathyroid hormone (PTH), and fibroblast growth factor 23 (FGF23), have been reported in patients with calcific aortic valve stenosis (CAVS). However, evidence of the causal role of mineral metabolism in CAVS is still lacking. In this study, we employed a systematic pipeline combining Mendelian randomization (MR), Steiger directionality test, colocalization analysis, protein-protein network, and enrichment analysis to investigate the causal effect of mineral metabolism on CAVS. Genome-wide association study (GWAS) and protein quantitative trait loci data for mineral metabolism markers were extracted from large-scale meta-analyses. Summary statistics for CAVS were obtained from two independent GWAS datasets as discovery and replication cohorts (*n* = 374,277 and 653,867). In MR analysis, genetic mimicry of serum FGF23 elevation was associated with increased CAVS risk [OR_discovery_ = 3.081 (1.649–5.760), *P*_discovery_ = 4.21 × 10^−4^; OR_replication_ = 2.280 (1.461 – 3.558), *P*_replication_ = 2.82 × 10^−4^] without evidence of reverse causation (*P*_steiger_= 7.21 × 10^−98^). Strong colocalisation association with CAVS was observed for FGF23 expression in the blood (PP.H4 = 0.96). Additionally, we identified some protein-protein interactions between FGF23 and known CAVS-associated genes. Serum calcium, phosphate, 25OH-VitD, and PTH failed to show causal effects on CAVS at Bonferroni-corrected significance (all *P* > 0.05/5 = 0.01). In conclusion, elevated serum FGF23 level may act as a causal risk factor for CAVS, and its mechanism of action in CAVS development may be independent of its function in regulating mineral metabolism. Hence, FGF23 may serve as a circulating marker and a promising preventive target for CAVS, warranting further investigation.

## Introduction

Calcific aortic valve stenosis (CAVS) is the most common form of valvular heart disease in the elderly, and its disease burden continues to increase as the population ages^1^. CAVS is characterized by progressive thickening and calcification of the valve leaflets, leading to obstruction of blood flow, heart failure, and eventual death^2^. Several hypotheses have been proposed to explain the pathogenesis of CAVS^3,4^. However, except for surgical valve replacement, no medical treatment is effective in preventing the progression of CAVS based on the existing mechanisms^5–7^. Furthermore, it remains a great challenge to identify individuals at high risk of CAVS progression.

Disorders of mineral metabolism have recently emerged as a risk factor for CAVS. The association between elevated serum levels of calcium and phosphate and CAVS has been extensively observed in patients with chronic kidney disease (CKD)^8–11^. However, in patients with preserved renal function, conflicting findings exist in the literature. Some studies have demonstrated a positive correlation between serum calcium or phosphate levels and the risk of CAVS^12–15^, while others have reported no such association^16,17^. Additionally, there are inconsistent findings concerning the association of CAVS with mineral metabolism regulators, including 25-hydroxyvitamin D (25OH-VitD)^14,16–19^, parathyroid hormone (PTH)^13,14,16–18^, and fibroblast growth factor 23 (FGF23)^17,18,20^. These inconsistencies may arise from the heterogeneity of the study population and inadequate control for residual confounders may contribute to inconsistent findings, which are inherent limitations of observational studies.

Mendelian randomization (MR) has been widely utilized to infer causality between exposures and disease risks, leveraging the increasing availability of genome-wide association studies (GWASs)^21^. In MR design, single nucleotide polymorphisms (SNPs) act as unconfounded instrumental variables (IVs) to proxy phenotypes, assessing whether carriers of genetic variants exhibit different disease risks than non-carriers. Participants in an MR study are effectively randomized based on the random assignment of genetic variants during meiosis, a process akin to randomized controlled trials (RCTs), which minimizes the potential for confounding factors to bias causal inference^21^. Furthermore, genotype formation precedes disease onset and is typically unaffected by disease progression, thereby reducing the likelihood of reverse causality^21^.

Therefore, in this study, we employed a systematic multi-step pipeline combining MR design, Steiger directionality test, Bayesian colocalization analysis, protein-protein interaction network, and functional enrichment analysis to explore the causal relationship between serum calcium, phosphate, 25OH-VitD, PTH, and FGF23 with CAVS.

## Results

### Instrumental variables selection

Eligible SNPs were carefully chosen following a series of steps in the genetic instrument selection processes. All chosen SNPs were required to be independent (LD r^2^ <0.001), and no palindromic SNPs or SNPs with incompatible alleles were detected. We further manually investigated the secondary traits of the mineral metabolism-associated SNPs to identify and exclude confounders that may invalidate the MR estimates. Among the 7 SNPs associated with serum calcium levels, rs780094 within the GCKR gene region had notable pleiotropic associations with lipid profiles, glycemic traits, type 2 diabetes, and adiposity (*P* < 5.00 × 10^−8^) (Supplementary Data 6). These metabolic disorder phenotypes might introduce confounding factors into the CAVS analysis^3,4^. Moreover, we found that rs1697421 and rs2769071, in addition to their associations with serum phosphate and FGF23, respectively, also showed significant correlations with alkaline phosphatase levels (*P* = 1.70 × 10^−14^ and 2.62 × 10^−51^, respectively). Notably, alkaline phosphatase is recognized as a potential risk factor for CAVS^22,23^. Consequently, the three aforementioned SNPs were excluded from subsequent analyses. The remaining SNPs were not found to be associated with common risk factors of CAVS (smoking, hypertension, diabetes mellitus, obesity, lipid metabolism disorder, and CKD)^3,4^. Additionally, two SNPs absent in the outcome GWAS datasets were replaced with proxy SNPs with high-LD (rs6727030 replacing rs1550532, *r*^2^ = 0.96; rs2144530 replacing rs8018720, *r*^2^ = 1, Supplementary Data 3).

In total, 22 SNPs were selected as IVs for the MR analysis, including six for calcium, three for phosphate, six for 25OH-VitD, three for PTH, and four for FGF23. These IVs explained 0.59% to 2.67% of the variance in the exposure traits, with F-statistics ranging from 29.16 to 1497.32, suggesting that instrument bias was improbable to affect the analyses. The strength of the IVs for each exposure trait and the statistical power of the MR analysis are presented in Supplementary Data 3 and Supplementary Data 4, respectively.

### Primary analysis

**Fig. 1:**
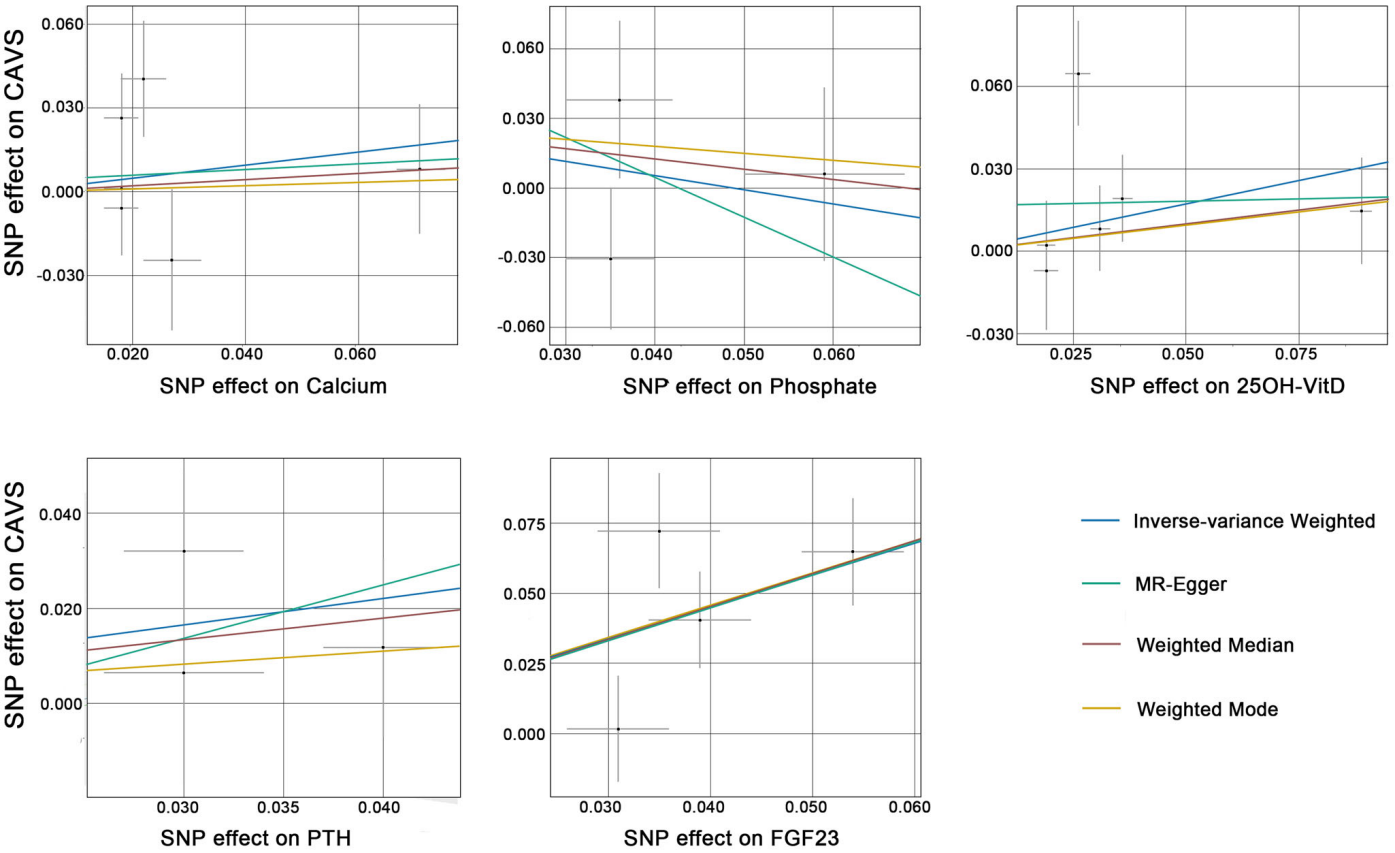
Scatterplot for the Mendelian randomization association between mineral metabolism markers and calcific aortic valve stenosis. CAVS calcific aortic valve stenosis, PTH parathyroid hormone, FGF23 fibroblast growth factor 23, SNP single nucleotide polymorphism.

In the IVW analysis, the genetically predicted circulating FGF23 concentration was significantly associated with the risk of CAVS. Specifically, with per 1-standard deviation increase in natural logarithm-transformed FGF23 concentration, the odds ratio (OR) of CAVS was 3.081 (95% confidence interval (CI) = 1.649–5.760, *P* = 4.21 × 10^−4^). However, no causal relationship was found between serum levels of calcium (OR = 1.270, 95%CI = 0.706 – 2.284, *P* = 0.42), phosphate (OR = 0.664, 95%CI = 0.344 – 1.283, *P* = 0.22), 25OH-VitD (OR = 1.409, 95%CI = 0.881 – 2.253, *P* = 0.15), or PTH (OR = 1.737, 95%CI = 0.924 – 3.266, *P* = 0.09) and CAVS at the Bonferroni-corrected significance (*P* > 0.05/5 = 0.01). The results of the primary analyses are presented in Table 1 and Fig. 1. Supplementary Fig. 1 shows the results of the Wald ratio method with a single SNP as an exposure tool.

**Table 1:** Mendelian randomization results

Exposure	Methods	Number of SNPs	Discovery Cohort			Replication Cohort		
			OR	95% CI	*P*	OR	95% CI	*P*
Calcium	Inverse-variance weighted	6	1.270	0.706 – 2.284	0.42	1.115	0.689 – 1.805	0.66
	Weighted median	6	1.115	0.631 – 1.970	0.71	1.008	0.606 – 1.676	0.98
	Weighted mode	6	1.056	0.561 – 1.987	0.87	0.987	0.579 – 1.682	0.96
	MR Egger	6	1.107	0.319 – 3.841	0.45	1.086	0.486 – 2.430	0.43
Phosphate	Inverse-variance weighted	3	0.664	0.344 – 1.283	0.22	0.662	0.374 – 1.171	0.16
	Weighted median	3	0.746	0.356 – 1.563	0.44	0.687	0.337 – 1.400	0.30
	Weighted mode	3	0.817	0.364 – 1.837	0.67	0.739	0.323 – 1.690	0.55
	MR Egger	3	0.355	0 – 19632.083	0.34	0.297	0.010 – 8.833	0.19
25OH-VitD	Inverse-variance weighted	6	1.409	0.881 – 2.253	0.15	0.824	0.499 – 1.361	0.45
	Weighted median	6	1.220	0.830 – 1.793	0.31	0.712	0.510 – 0.994	0.05
	Weighted mode	6	1.210	0.821 – 1.783	0.38	0.704	0.509 – 0.974	0.09
	MR Egger	6	1.002	0.529 – 1.900	0.49	0.543	0.321 – 0.918	0.01
PTH	Inverse-variance weighted	3	1.737	0.924 – 3.266	0.09	1.650	0.931 – 2.925	0.09
	Weighted median	3	1.566	0.768 – 3.195	0.22	1.818	0.983 – 3.361	0.06
	Weighted mode	3	1.317	0.543 – 3.193	0.60	1.825	0.886 – 3.760	0.24
	MR Egger	3	2.919	0.301 – 28.282	0.17	3.168	0.470 – 21.356	0.12
FGF23	Inverse-variance weighted	4	3.081	1.649 – 5.760	4.21 × 10^−4^	2.280	1.461 – 3.558	2.82 × 10^−4^
	Weighted median	4	3.091	1.733 – 5.514	1.76 × 10^−4^	2.625	1.530 – 4.502	4.20 × 10^−4^
	Weighted mode	4	3.138	1.639 – 6.006	0.04	2.664	1.399 – 5.071	0.06
	MR Egger	4	3.263	0.326 – 32.674	0.15	2.091	0.833 – 5.248	0.05

*SNP* single nucleotide polymorphism, *OR* odds ratio, *CI* confidence interval, *25OH-VitD* 25-hydroxyvitamin D, *PTH* parathyroid hormone, *FGF23* fibroblast growth factor 23.

### Sensitivity analyses

In complementary analyses, the MR estimates obtained from the weighted median (OR = 3.091, 95%CI = 1.733 – 5.514, *P* = 1.76 × 10^−4^), the weighted mode (OR = 3.138, 95%CI = 1.639 – 6.006, *P* = 0.04) and the MR-Egger (OR = 3.263, 95%CI = 0.326 – 32.674, *P* = 0.15) demonstrated consistent directionality and magnitude with the primary analysis, supporting the robustness of the causal relationship between FGF23 and CAVS. Notably, no heterogeneity was detected based on Cochran’s Q test and Rucker’s Q test (all *P* > 0.05/5 = 0.01) (Supplementary Data 5). Moreover, the MR-Egger regression intercept, falling within a range close to zero (0.012 to 0.059) with all *P* > 0.05/5 = 0.01, indicated no significant evidence of horizontal pleiotropy (Supplementary Data 5). Moreover, the LOO analysis did not identify any high-influential SNPs that might introduce bias to the pooled effect estimates (Supplementary Fig. 2).

### Replication and Meta-analysis

As depicted in Table 1, in replication analyses, FGF23 remained to be a crucial causal factor of CAVS (OR = 2.280, 95%CI = 1.461 – 3.558, *P* = 2.82 × 10^−4^), whereas serum calcium (OR = 1.115, 95%CI = 0.689–1.805, *P* = 0.66), phosphate (OR = 0.662, 95%CI = 0.374–1.171, *P* = 0.16), 25OH-VitD (OR = 0.824, 95%CI = 0.499 – 1.361, *P* = 0.45), or PTH (OR = 1.650, 95%CI = 0.931 – 2.925, *P* = 0.09) were not found to be causal.

**Fig. 2:**
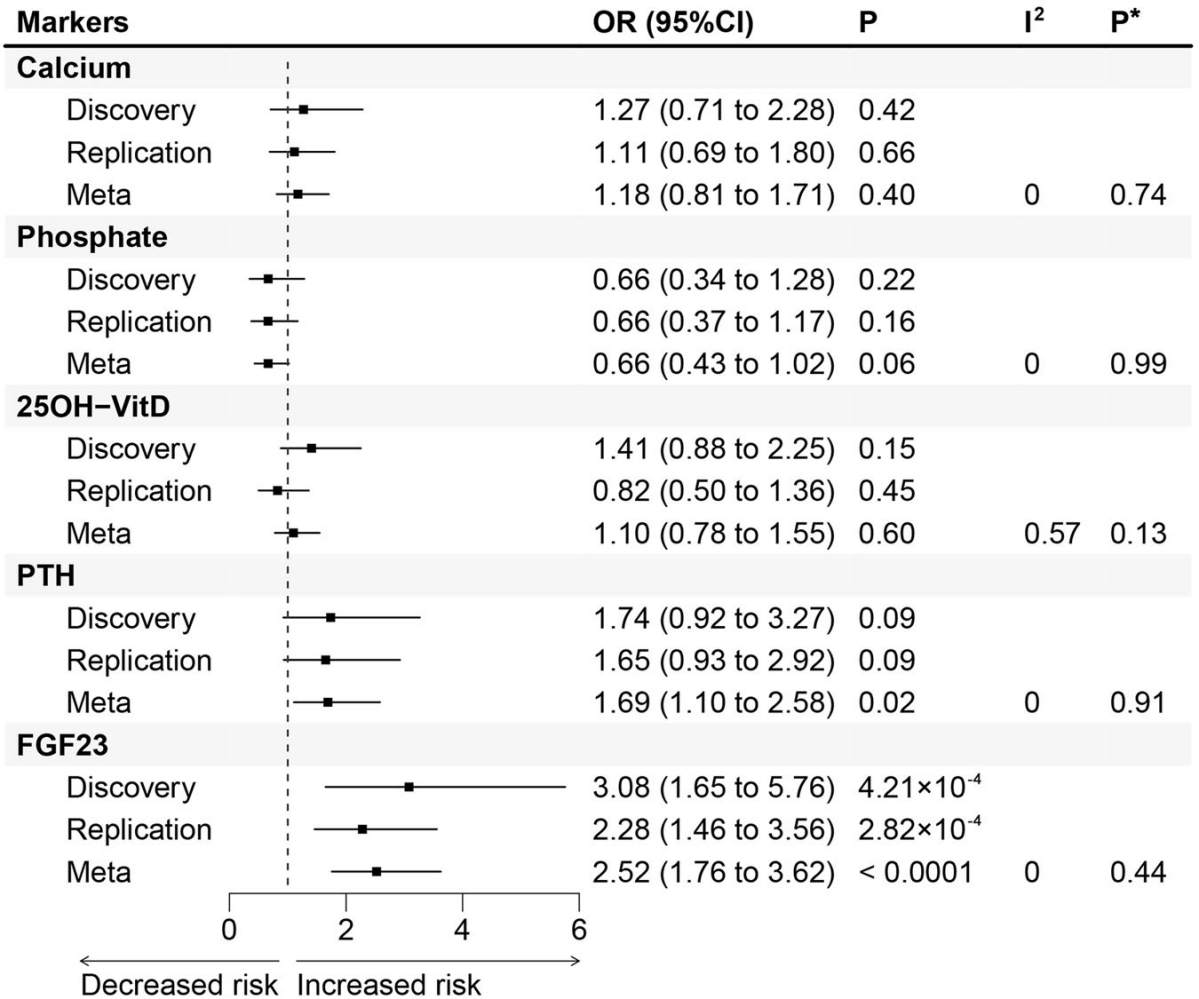
Meta‑analysis of the causal effect of mineral metabolism markers on the risk of calcific aortic valve stenosis derived from inverse-variance weighted. OR odds ratio, CI confidence interval, PTH parathyroid hormone, FGF23 fibroblast growth factor 23. P* indicates *P* for heterogeneity test in Meta-analysis.

In the Meta-analysis, which combined the discovery and replication cohorts into a pooled dataset, a robust causal relationship between FGF23 and CAVS was further identified (OR = 2.523, 95%CI = 1.756 – 3.625, *P* < 1.00 × 10^−4^). Conversely, serum calcium (OR = 1.175, 95%CI = 0.810 – 1.705, *P* = 0.40), phosphate (OR = 0.663, 95%CI = 0.431 – 1.020, *P* = 0.06), 25OH-VitD (OR = 1.097, 95%CI = 0.779 – 1.546, *P* = 0.60), and PTH (OR = 1.689, 95%CI = 1.105 – 2.581, *P* = 0.02) failed to show a causal relationship with CAVS at the Bonferroni-corrected significance (*P* > 0.05/5 = 0.01) (Fig. 2).

### Direction validation and colocalization analysis

**Fig. 3:**
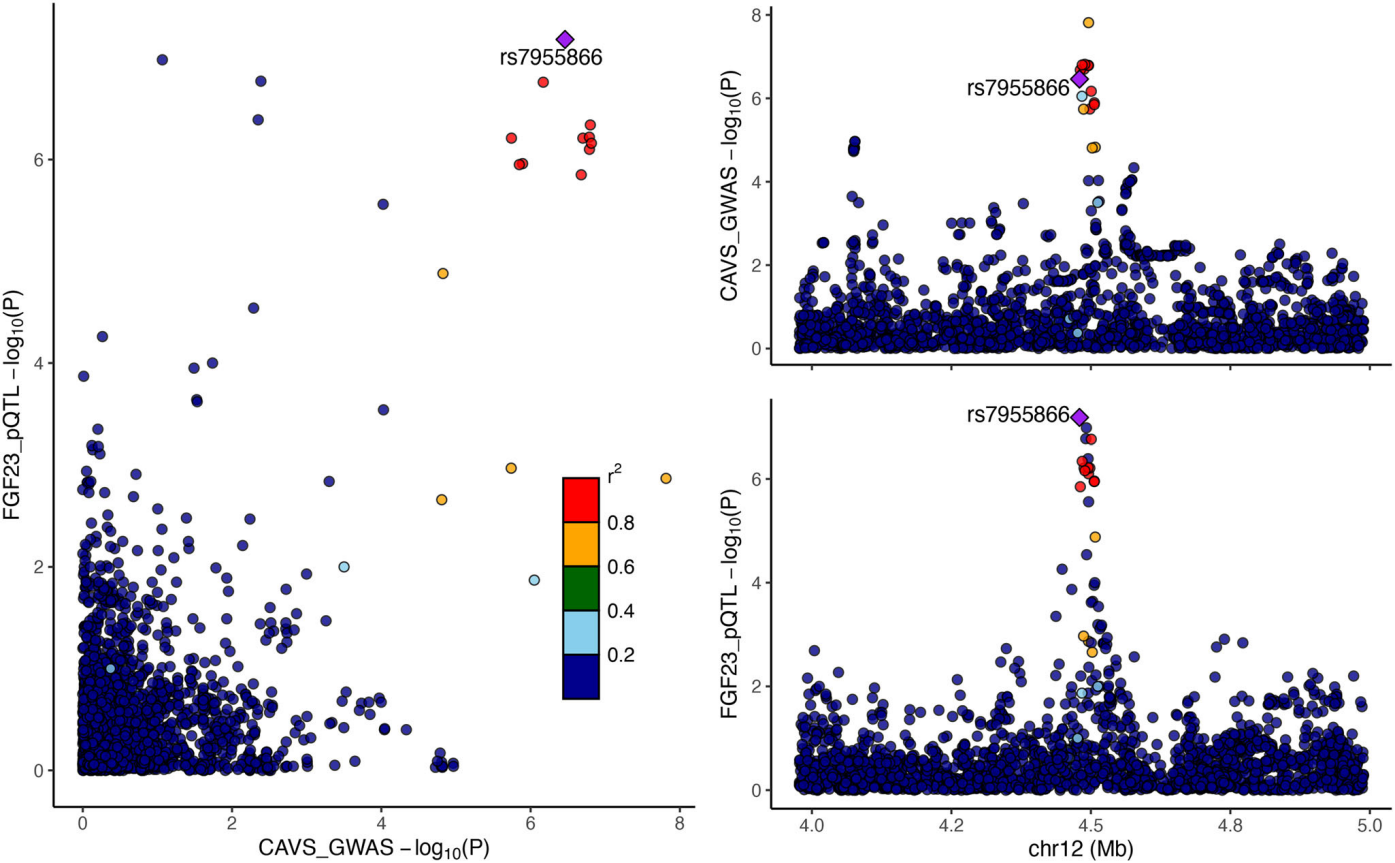
Bayesian colocalization analysis of the protein quantitative trait loci (pQTL) for FGF23 expression in the blood and calcific aortic valve stenosis (CAVS) risk. FGF23 fibroblast growth factor 23, GWAS genome-wide association study.

We conducted a Steiger test to validate the directional effect from FGF23 to CAVS. The Steiger test yielded a *p*-value of 7.21 × 10^−98^, robustly supporting the absence of bias from reverse causation in the identified causal relationship and indicating genuine causality. To further identify the probability that genetic variants associated with FGF23 expression levels in the blood and CAVS shared causal SNPs, Bayesian colocalization analysis was performed. As illustrated in Fig. 3, FGF23 expression in the blood and CAVS shared a causal variant (PP.H4 = 96%), providing additional evidence supporting FGF23 as a potential causal protein for CAVS.

### Protein-protein interaction network and functional enrichment analysis

**Fig. 4:**
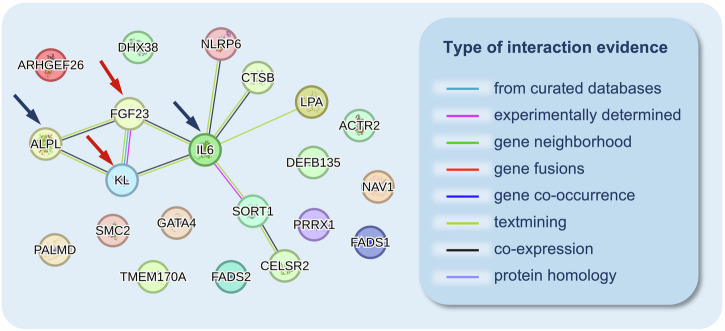
The protein-protein interaction network using Mendelian randomization-identified proteins passed multiple corrections and calcific aortic valve stenosis-associated genes. The red arrow indicates FGF23 and its co-receptor klotho. The blue arrow indicates the established calcific aortic valve stenosis-associated genes.

To explore the interactions between FGF23 and the established associated genes for CAVS, we conducted a protein-protein interaction network analysis using the STRING database. Our investigation revealed that FGF23 and its co-receptor Klotho both exhibit co-expression interactions with ALPL and IL-6 (Fig. 4). Furthermore, through functional enrichment analysis, we identified several plausible biological processes that may be involved in the association of FGF23 with ALPL and IL-6. (Supplementary Data 9)

## Discussion

In this MR analysis involving 22,918 CAVS cases and 1,008,226 controls, we revealed a novel and significant causal relationship between elevated serum FGF23 levels and an increased risk of CAVS. This discovery was validated by two independent GWAS datasets for CAVS, and a meta-analysis of their results further affirmed the findings. Additionally, the directionality of the effect was confirmed through the Steiger directionality test, and colocalization analysis using serum FGF23 pQTL data provided further validation at the protein expression level. Importantly, our findings did not reveal evidence regarding a causal effect of calcium, phosphate, 25OH-VitD, and PTH on the incidence of CAVS, suggesting that the mechanism of action of FGF23 in CAVS development may be independent of its role in regulating mineral homeostasis.

Based on the available clinical evidence, FGF23 appears to be a causal risk factor for CAVS. Kirkpantur first reported significantly higher FGF23 levels in dialysis patients with CAVS identified by echocardiography compared to those without^24^. Linefsky et al. further evaluated phosphate metabolism markers and CAVS incidence in a community-based prospective cohort enrolling 5145 multi-ethnic individuals without CT-assessed CAVS at baseline^18^. Over a mean follow-up of 2.4 years, 4.1% of patients were newly diagnosed with CAVS, and the highest FGF23 quartile was the only marker independently associated with an increased incidence of CAVS^18^. On the other hand, it has been proven that cinacalcet can significantly lower serum FGF23 levels^25^. The ADVANCED study, a randomized controlled trial, revealed that treatment with cinacalcet plus low-dose vitamin D significantly decelerated the progression of CAVS compared to treatment with flexible doses of vitamin D alone in patients receiving hemodialysis^26^. The aforementioned bidirectional evidence from populations with elevated and reduced FGF23 strongly suggests a positive association of FGF23 with the incidental risk of CAVS. Our study further supports this causal relationship using the MR design, effectively addressing biases inherent in observational studies, including limited sample sizes, reverse causality, and residual confounding^21^. Therefore, FGF23 is a promising target for preventing CAVS using therapies such as cinacalcet or specific antibodies^25^.

While the association between FGF23 and CAVS is well-established, the underlying mechanisms remain incompletely understood. FGF23, primarily produced by osteocytes in the bone, regulates serum phosphate and vitamin D metabolism^27^. However, its adverse effects on the cardiovascular system have gained increasing attention. Elevated FGF23 levels have been associated with increased mortality rates in patients with both CKD^28,29^ and normal renal function^30^. Moreover, elevated FGF23 levels are correlated with increased endothelial dysfunction^31^, arterial stiffness^31^, total body atherosclerotic burden^32^, left ventricular hypertrophy^33^, and an increased risk of major adverse cardiovascular events^30^. These associations appear robust and independent of other concomitant abnormalities in mineral metabolism, as evidenced in patients with normal renal function^34^. In this context, various mechanisms beyond mineral metabolism have been proposed to elucidate the role of FGF23 in CAVS. Firstly, elevated FGF23 levels can directly activate the renin-angiotensin-aldosterone system (RAAS)^35^, thereby fostering the development of CAVS through mechanisms involving monocyte infiltration, inflammatory cytokine production, and osteogenic differentiation of valvular interstitial cells^36^. Additionally, FGF23 can induce endothelial dysfunction^31^, disrupting nitric oxide expression and promoting calcium deposition, thereby contributing to CAVS^37^. Moreover, our protein-protein interaction network analysis identified that FGF23 could interact with the established CAVS-associated genes, such as ALPL and IL-6, suggesting that FGF23 may be involved in the pathogenesis of CAVS through known pathways. Nevertheless, direct experimental evidence is still lacking and warrants further investigation.

The relationship between serum calcium, phosphate levels, and CAVS remains controversial. Notably, CAVS is frequently observed in patients with CKD, who often manifest with abnormalities in serum calcium and phosphate metabolism. Previous studies have noted this association and consequently attributed CAVS to elevated calcium and phosphate levels^8–11^. However, in individuals with preserved renal function, the association between serum calcium, phosphate levels, and CAVS appears conflicting. Some studies reported a positive association with both serum calcium and phosphate^14,15^, some studies reported a positive association with either serum calcium only^12,13^ or serum phosphate only^19,38^, while others reported no association with either^16,17^. Furthermore, observational studies exploring the correlation between CAVS incidence and mineral regulators, including vitamin D^14,16–19^ and PTH^13,14,16–18^, have also yielded discrepant results.

Despite several proposed mechanisms explaining the potential involvement of mineral metabolism in the development of CAVS^8^, it remains unclear whether serum calcium, phosphate, and mineral regulators are causative factors or merely epiphenomena for CAVS. As demonstrated by Linefsky et al., serum phosphate levels were only associated with the baseline prevalence of CAVS in the community population^18^. However, among those without CAVS at baseline, there was no significant association of serum phosphate with incident CAVS during follow-up^18^. Our results provide a novel perspective for explaining the existing contradictions. In this study, a robust causal link between serum FGF23 and CAVS was evident, while serum calcium, phosphate, and related regulatory hormones showed no associations. As a sensitive marker of renal dysfunction, serum FGF23 levels rise before serum phosphate and creatinine levels become abnormal^39^, whereas previous observational studies have lacked adjustment for FGF23 in their multivariate analyses. Hence, FGF23 may act as a significant confounding factor, potentially leading to a spurious relationship between mineral metabolism and CAVS.

Several limitations should be considered when interpreting our study. Firstly, variations in mineral metabolism marker concentrations may have diverse effects on patients with CAVS of different ages, genders, or comorbidities. However, the absence of individual-level information in the summary statistics prevents us from stratifying CAVS by age, gender, and comorbidities. Secondly, it should be noted that the GWASs on CAVS did not classify the severity of CAVS. Therefore, further research is needed to estimate the precise impact of FGF23 levels on the severity of CAVS. Thirdly, while our replication cohort is derived from the largest-scale GWAS meta-analysis for CAVS, the constituent cohorts employed various diagnostic criteria, including echocardiography, international classification of disease codes, and aortic valve replacement surgeries. However, the robustness of the findings was verified in the FinnGen Consortium with consistent diagnostic criteria and the pooled dataset of the Meta-analysis. Fourthly, as a potential confounder affecting mineral metabolism and valve calcification, CKD was not excluded from the GWAS datasets for calcium, 25OH-VitD, and CAVS. Although the SNPs used as IVs in the MR analysis were not found to be associated with CKD, and various sensitivity analyses were conducted to test the assumptions of the MR analysis, horizontal pleiotropy cannot be eliminated entirely. Fifthly, despite utilizing extensive GWAS datasets to extract genetic variants, IVs could only explain a small portion of trait variance, leading to reduced statistical power for detecting modest associations with numerous mineral metabolism markers. Thus, these analyses should be repeated when more extensive GWAS data becomes accessible. Finally, given that the GWAS in this study was conducted on the European population, the generalizability of our findings to non-European populations remains uncertain.

In conclusion, this MR study suggests that elevated serum FGF23 level may be a causal risk factor for CAVS. There is no significant causal effect of serum calcium, phosphate, 25OH-VitD, and PTH on CAVS, implying that the mechanism of action of FGF23 in CAVS development might be independent of its function in regulating mineral metabolism. Hence, FGF23 could potentially serve as a circulating marker for predicting CAVS and could be a promising target for preventive interventions. Nonetheless, it’s essential to recognize the limitations of this study, and further investigation into the underlying mechanisms of FGF23 in CAVS pathogenesis is warranted to provide a more comprehensive understanding.

## Methods

### Study design

We used a two-sample MR design to assess the potential causal relationship between serum levels of calcium, phosphate, 25OH-VitD, PTH, and FGF23 and the risk of CAVS. Following Mendel’s second law, genetic variants (alleles) undergo random assignment during meiosis. Therefore, participants in an MR study are ‘randomized’ according to the presence of alleles. This is similar to RCTs, allowing inferences on the effect of mineral metabolism on CAVS risk without further adjustment for confounders^21^. This study adhered to the three fundamental assumptions of the MR approach: (1) Relevance: genetic instruments should be strongly correlated with exposure; (2) Independence: genetic instruments are independent of the confounders influencing the relationship between exposure and outcome; (3) Exclusivity: genetic instruments influence the outcome only through the exposures of interest^21^. Genetic information for all exposure and outcome traits was derived from large-scale, non-overlapping GWAS consortia comprising individuals of European ancestry. These GWASs have adjusted for covariates including age, sex, ancestry, and study site to avoid confounding effects from demographic factors (Supplementary Data 2). The causal direction was confirmed through the Steiger directionality test. Further validation of the MR findings was performed using Bayesian colocalization analysis based on summary-level data from genome-wide protein quantitative trait loci (pQTL) studies. Protein-protein interaction network and functional enrichment analysis were used to explore the potential relationship between the MR-identified CAVS risk factors and the known associated genes of CAVS.

**Fig. 5:**
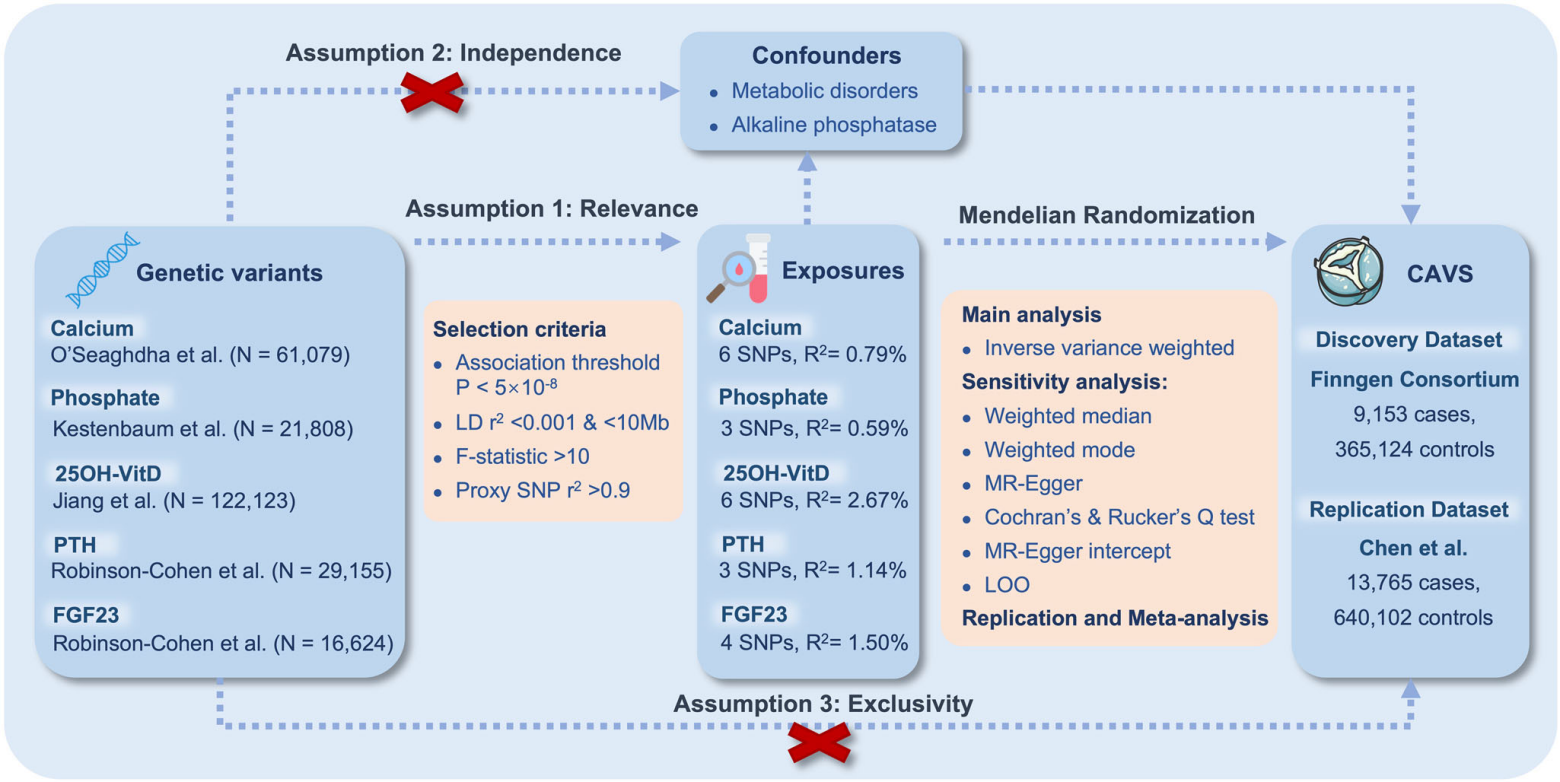
Overview of the Mendelian randomization design. Assumption 1: genetic instruments should be strongly correlated with exposure; Assumption 2: genetic instruments are independent of the confounders influencing the relationship between exposure and outcome; Assumption 3: genetic instruments influence the outcome only through the exposures of interest. 25OH-VitD 25-hydroxyvitamin D, PTH parathyroid hormone, FGF23 fibroblast growth factor 23, LD linkage disequilibrium, SNP single nucleotide polymorphism, LOO Leave-one-out, CAVS calcific aortic valve stenosis, GWAS genome-wide association study.

This study adhered to the Strengthening the Reporting of Observational Studies in Epidemiology-Mendelian Randomization (STROBE-MR) reporting guidelines (detailed information in Supplementary Data 1)^40^, and its design is illustrated in Fig. 5. Ethical approval was not required for the present study, as all data sources were based on publicly available data; specific ethical review and informed consent had been obtained in the original studies.

### GWAS data for calcium, phosphate, 25OH-VitD, PTH, and FGF23

We selected the genome-wide significant SNPs (*P* < 5.00 × 10^−8^) associated with serum levels of calcium^41^, phosphate^42^, 25OH-VitD^43^, PTH^44^, and FGF23^45^ from the most recent large-scale GWAS meta-analyses. The chosen studies were all based on European ancestry, including 61,079 individuals for calcium, 21,808 for phosphate, 122,123 for 25OH-VitD, 29,155 for PTH, and 16,624 for FGF23. Additionally, participants with an estimated glomerular filtration rate (eGFR) <45 ml/min/1.73 m^2^ in the phosphate dataset and eGFR <30 ml/min/1.73 m^2^ in the PTH and FGF23 dataset have been excluded to avoid confounders of renal dysfunction^42,44,45^. Detailed information regarding the data sources, sample sizes, adjusted factors, and significant SNPs of the included GWAS datasets can be found in Supplementary Data 2.

### GWAS data for CAVS

For the discovery analyses, summary genetic association data for CAVS was extracted from Freeze 9 of the FinnGen Consortium, involving 9153 cases and 368,124 controls of European ancestry^46^. CAVS cases were defined as participants with a diagnosis code of ICD-9 424.1, ICD-10 I35.0 or I35.2, or NOMESCO Classification of Surgical Procedures codes for aortic valve repair or replacement (codes FMA, FMB, FMD, and FME) in hospital inpatient records^46^. To validate our findings through replication analysis, we used GWAS data for CAVS from a large-scale GWAS meta-analysis, comprising 13,765 cases and 640,102 controls from 10 European ancestry cohorts^47^. The diagnostic criteria for CAVS, demographic factors, and quality control procedures of each cohort were detailed in the original GWAS study^47^. No duplication of cohorts was found between these two GWAS datasets (Supplementary Data 2).

### pQTLs data

For the mineral metabolism markers that demonstrated significance for the risk of CAVS in the MR analysis, the corresponding summary-level pQTLs data were obtained from the SCALLOP Consortium for further validation through Bayesian colocalization analysis^48^. pQTLs are genetic variants associated with protein expression levels. The SCALLOP Consortium recruited 14,824 participants of European ancestry from 11 cohorts and performed genome-wide pQTL mapping for 91 plasma proteins measured using the Olink Target panel (Supplementary Data 2)^48^.

### Genetic instrument selection

A series of procedures were used to select eligible IVs from the exposure SNPs with genome-wide significance (*P* < 5.00 × 10^−8^). Firstly, to mitigate bias from linkage disequilibrium (LD), LD analyses were performed to ensure that SNPs were independent, using a threshold of *r*^2^ < 0.001 within a physical distance of 10 kb. Secondly, to avoid bias caused by weak instruments, F-statistics were calculated for each SNP to measure statistical strength using mRnd (http://cnsgenomics.com/shiny/mRnd/). SNPs with F-statistic <10 were considered weak instruments and subsequently excluded. Thirdly, to avoid violating the MR independence assumptions, the horizontal pleiotropy of each proposed IV was carefully evaluated using Phenoscanner (http://www.phenoscanner.medschl.cam.ac.uk/). We also manually investigated whether the secondary traits of the mineral metabolism-associated SNPs were meanwhile associated with several common risk factors of CAVS that might bias the MR estimates, including smoking, hypertension, diabetes mellitus, obesity, lipid metabolism disorder, and CKD^3,4^. Fourthly, to guarantee the MR exclusivity assumption, we extracted the exposure SNPs from the outcome GWAS datasets and excluded those associated with CAVS (*P* < 5.00 × 10^−8^). For SNPs absent in the outcome cohorts, robust proxy SNPs were identified based on the European reference panel of the 1000 Genomes Project (http://snipa.org/snipa3/). Only proxies showing strong LD with *r*^2^ > 0.9 were adopted, a much stricter criterion than that commonly used in other MR analyses (i.e., *r*^2^ > 0.8); otherwise, they were discarded. Then, a harmonization process was performed to align the alleles of exposure and outcome SNPs and to exclude palindromic and incompatible SNPs. Supplementary Data 3 provides details of the SNPs used for MR analysis.

### Statistical analyses

#### Two-sample Mendelian randomization analysis

The random-effect inverse-variance weighted (IVW) method served as the primary analysis to obtain MR estimates. As a widely used method in MR analysis, IVW combines all Wald ratios from individual SNPs to generate a pooled estimate and assumes that all the genetic variants are valid^49^, making it the most powerful tool for MR estimation. Therefore, IVW was utilized as the primary method to explore preliminary causal associations between the exposures and the outcome.

Sensitivity analyses were conducted to evaluate potential bias related to the MR assumptions^50^. We applied the weighted median, weighted mode, and MR-Egger (slope term, with bootstrap to estimate standard errors) as complementary methods for MR analysis^51–53^. To identify the presence of heterogeneity, the Cochran’s Q test (IVW) and Rucker’s Q test (MR-Egger) were employed^54^. Horizontal pleiotropy was evaluated using the MR-Egger intercept^51^. The leave-one-out (LOO) analysis was performed to detect high-influential points that could significantly impact the pooled IVW estimates.

Consequently, the causal relationships were considered valid only when the following criteria were met: (1) consistent directions and magnitudes across all four MR methods; (2) absence of heterogeneity or pleiotropy; (3) no influential points were detected in the LOO analysis.

#### Replication and Meta-analysis

To validate the robustness of the findings obtained in the discovery cohort, we replicated the IVW, weighted median, and weighted mode analyses using an independent GWAS dataset^47^. Furthermore, we performed a meta-analysis and synthesized the results from the discovery and replication cohorts to estimate the overall causal effects. Heterogeneous effects in the Meta-analysis were identified using the I-statistic.

#### Causal direction validation

We used the Steiger directionality test to ensure that the observed causalities were not biased by reverse causation^55^. Specifically, if a combination of the included SNPs contributed more to the genetic risk of CAVS than the mineral metabolism markers (i.e., Steiger test *P* > 0.05), it indicated a potential bias in the direction of causal inference.

#### Bayesian colocalization analysis

To validate the exclusivity assumption and ascertain whether the given mineral metabolism marker and CAVS share common genetic causal variants, we employed Bayesian colocalization analysis^56^. Given that pQTLs are more predictive of gene functions than eQTLs^57^, we used pQTLs data in the Bayesian colocalization analysis. Specifically, Bayesian colocalization analysis provides five posterior probabilities of hypotheses (PPH) regarding whether a single variant is shared between the two traits: (1) PP.H0: no association with either trait; (2) PP.H1: a genetic variant only associated with the exposure, but not with the outcome; (3) PP.H2: a genetic variant associated with the outcome, but not with the exposure; (4) PP.H3: association with both the exposure and the outcome, with different causal variants; (5) PP.H4: association with the exposure and the outcome, with a shared causal variant^56^. The exposure and outcome traits are considered to share common variants if the PP. H4 > 80%.

#### Protein-protein interaction network and functional enrichment analysis

To explore the potential relationship between the MR-identified CAVS risk factors in this study and the established CAVS-associated genes, we conducted a protein-protein interaction network analysis using the Search Tool for the Retrieval of Interacting Genes (STRING) database (version 12.0, https://string-db.org/)^58^. The STRING database contains a wide range of protein-protein interactions, including direct (physical) and indirect (functional) associations. These interactions are derived from computational prediction, knowledge transfer between organisms, and interactions aggregated from other databases^58^. The CAVS-associated genes used in this analysis were obtained from the latest review and the largest-scale GWAS meta-analysis for CAVS (Supplementary Data 8)^3,47^. Additionally, to gain further insights into the potential pathways and biological processes involving the interacting proteins, we conducted functional enrichment analysis based on the Gene Ontology database incorporated within the STRING database^58^.

In this study, the Bonferroni-corrected significance level was used with a threshold of *P* < 0.01 (0.05/5) to account for multiple testing issues. All statistical analyses were conducted using the “TwoSampleMR” (version 0.5.7), “meta” (version 6.5-0), and “coloc” (version 5.2.3) packages in the R software environment (version 4.3.1).

## Supplementary information

Description of additional information

Supplementary Data

Supplementary information

## Data Availability

All data are publicly available. Detailed information for these datasets is summarized in Supplementary Data 2.
