# Supplementary material for: Genetic association of serum calcium, phosphate, vitamin D, parathyroid hormone, and FGF23 with the risk of aortic stenosis
Source: NPJ Cardiovasc Health. 2024 Aug 21;1:13. doi: 10.1038/s44325-024-00013-x (PMC12912326; doi:10.1038/s44325-024-00013-x)
Supplement: Supplementary file 1 — Description of additional information [file 44325_2024_13_MOESM1_ESM.pdf]

Supplementary Data

Supplementary Data 1: STROBE-MR checklist of recommended items to address in reports of mendelian randomization studies. CAVS, calcific aortic valve stenosis; 25OH-VitD, 25-hydroxyvitamin D; PTH, parathyroid hormone; FGF23, fibroblast growth factor 23; GWAS, genome-wide association study; pQTL, protein quantatitive trait loci; MR, mendelian randomization; SD, standard deviation.

Supplementary Data 2: Characteristics of genome-wide association studies and protein quantitative trait loci study used in mendelian randomization analyses. SNP, single nucleotide polymorphism; 25OH-VitD, 25-hydroxyvitamin D; PTH, parathyroid hormone; FGF23, fibroblast growth factor 23; CAVS, calcific aortic valve stenosis; pQTL, protein quantatitive trait loci; MR, mendelian randomization.

Supplementary Data 3: Details of instrumental variables used in mendelian randomization analyses. SNP, single nucleotide polymorphism; EAF, effect allele frequency; SE, standard error; 25OH-VitD, 25-hydroxyvitamin D; PTH, parathyroid hormone; FGF23, fibroblast growth factor 23.

Supplementary Data 4: Statistical power estimates for mendelian randomization analyses. OR, odds ratio; SNP, single nucleotide polymorphism; CAVS, calcific aortic valve stenosis; 25OH-VitD, 25-hydroxyvitamin D; PTH, parathyroid hormone; FGF23, fibroblast growth factor 23; GWAS, genome-wide association study.

Supplementary Data 5: Heterogeneity and pleiotropy tests of instrument effects. SE, standard error; 25OH-VitD, 25-hydroxyvitamin D; PTH, parathyroid hormone; FGF23, fibroblast growth factor 23.

Supplementary Data 6: SNPs excluded due to horizontal pleiotropy and their related phenotypes. SNP, single nucleotide polymorphism; SE, standard error.

Supplementary Data 7: Colocalisation results for FGF-23 expression in blood with the risk of calcific aortic valve stenosis risk. FGF23, fibroblast growth factor 23; pQTL, protein quantatitive trait loci; CAVS, calcific aortic valve stenosis.

Supplementary Data 8: Known calcific aortic valve stenosis associated genes. SNP, single nucleotide polymorphism.

Supplementary Data 9: Potential pathways and biological processes involved in the interaction of of FGF-23 with ALPL and IL-6.
