# Supplementary material for: Genetic association of serum calcium, phosphate, vitamin D, parathyroid hormone, and FGF23 with the risk of aortic stenosis
Source: NPJ Cardiovasc Health. 2024 Aug 21;1:13. doi: 10.1038/s44325-024-00013-x (PMC12912326; doi:10.1038/s44325-024-00013-x)

Supplementary Figures

Supplementary Figure 1 Forest plot for the causal effect of the single SNP on the risk of calcific aortic valve stenosis derived from inverse-variance weighted. OR, odds ratio; CI, confidence interval; 25OH-VitD, 25-hydroxyvitamin D; PTH, parathyroid hormone; FGF23, fibroblast growth factor 23; IVW, inverse-variance weighted.

Supplementary Figure 2. Forest plot for the Mendelian randomization leave-one-out analysis of the inverse-variance weighted estimates. OR, odds ratio; CI, confidence interval; 25OH-VitD, 25-hydroxyvitamin D; PTH, parathyroid hormone; FGF23, fibroblast growth factor 23; IVW, inverse-variance weighted.

Supplementary Figure 1. Forest plot for the causal effect of the single SNP on the risk of calcific aortic valve stenosis derived from inverse-variance weighted.

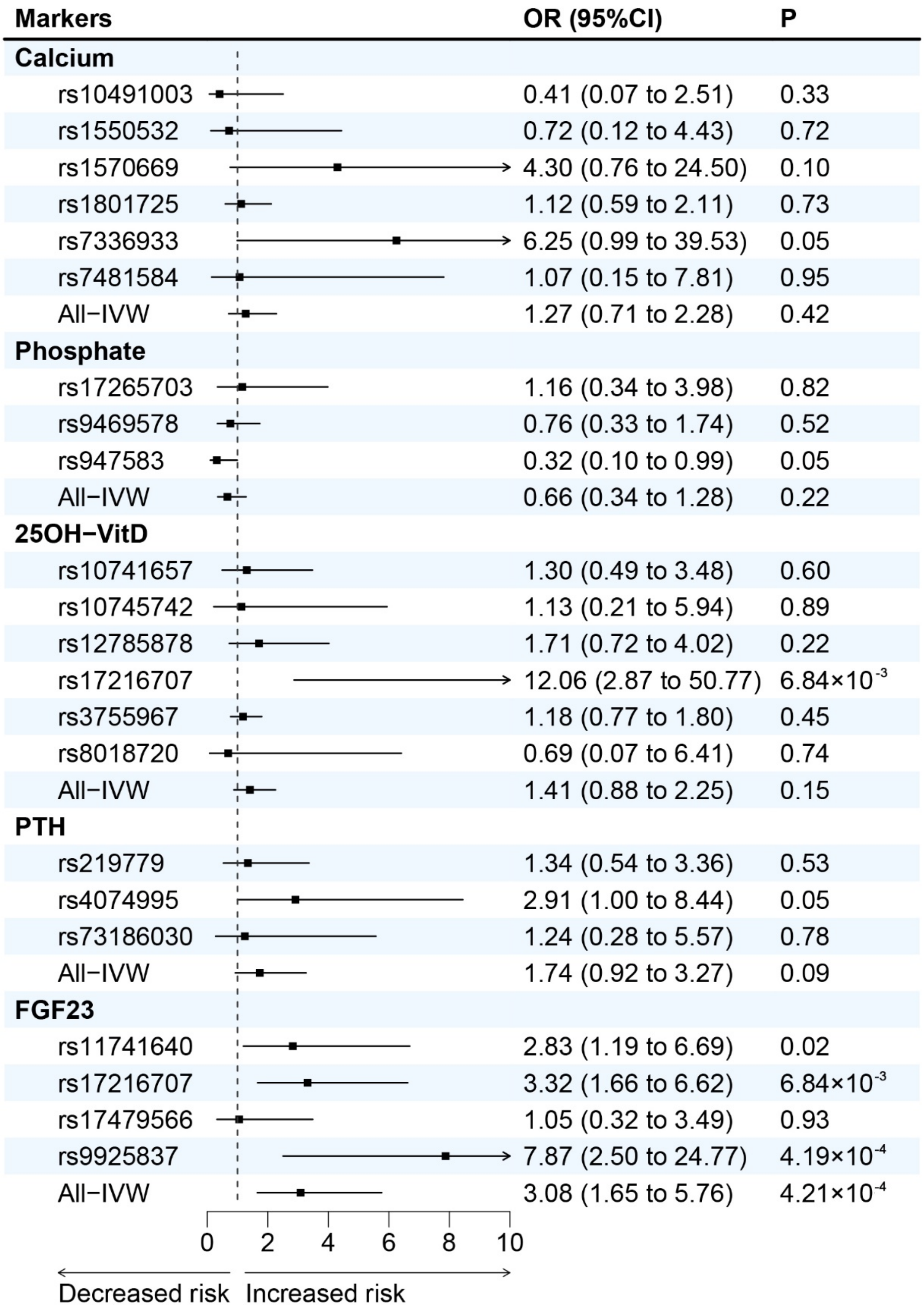

Supplementary Figure 2. Forest plot for the Mendelian randomization leave-one-out analysis of the inverse-variance weighted estimates.

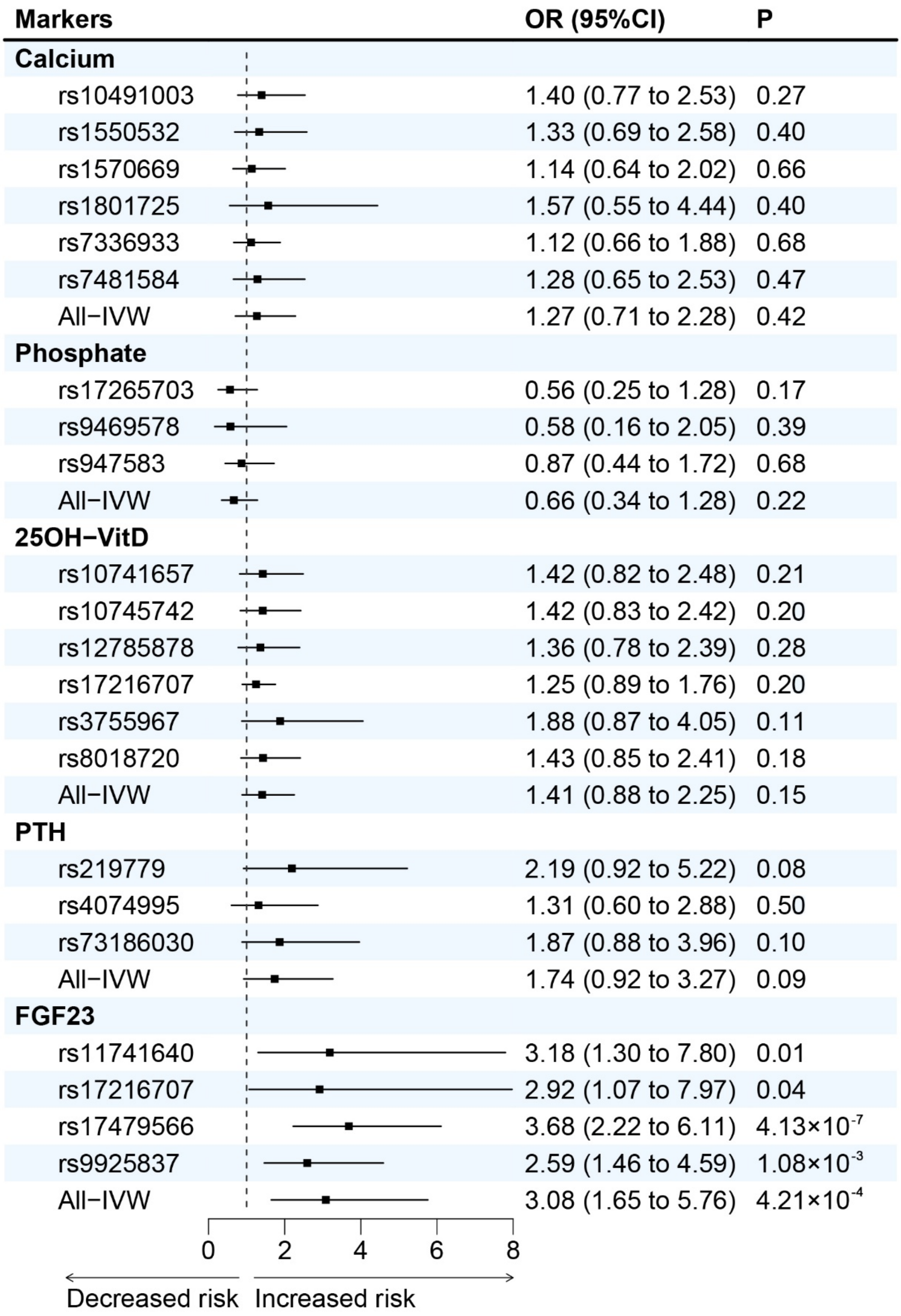

Supplement: Supplementary file 3 — Supplementary information [file 44325_2024_13_MOESM3_ESM.pdf]
